# Sweep-Out of Tigecycline, Chlortetracycline, Oxytetracycline, and Doxycycline from Water by Carbon Nanoparticles Derived from Tissue Waste

**DOI:** 10.3390/nano12203617

**Published:** 2022-10-15

**Authors:** Rasmiah S. Almufarij, Babiker Y. Abdulkhair, Mutaz Salih, Nujud M. Alhamdan

**Affiliations:** 1 Department of Chemistry, College of Science, Princess Nourah Bint Abdulrahman University, P.O. Box 84428, Riyadh 11671, Saudi Arabia; 2 Chemistry Department, College of Science, Imam Mohammad Ibn Saud Islamic University (IMSIU), P.O. Box 90905, Riyadh 11623, Saudi Arabia; 3 Chemistry Department, Faculty of Science, Sudan University of Science and Technology (SUST), Khartoum P.O. Box 13311, Sudan; 4 Department of Chemistry, College of Science and Humanities-Hurrymilla, Imam Mohammad Ibn Saud Islamic University (IMSIU), P.O. Box 5701, Riyadh 11432, Saudi Arabia

**Keywords:** carbon nanoparticles, toilet paper, tigecycline, chlortetracycline, oxytetracycline, doxycycline

## Abstract

Pharmaceutical pollution has pervaded many water resources all over the globe. The propagation of this health threat drew the researchers’ concern in seeking an efficient solution. This study introduced toilet paper waste as a precursor for carbon nanoparticles (CRNPs). The TEM results showed a particle size range of 30.2 nm to 48.1 nm, the BET surface area was 283 m^2^ g^−1^, and the XRD pattern indicated cubical-graphite crystals. The synthesized CRNPs were tested for removing tigecycline (TGCN), chlortetracycline (CTCN), oxytetracycline (OTCN), and doxycycline (DXCN) via the batch process. The adsorption equilibrium time for TGCN, DXCN, CTCN, and OTCN was 60 min, and the concentration influence revealed an adsorption capacity of 172.5, 200.1, 202.4, and 200.0 mg g^−1,^ respectively. The sorption of the four drugs followed the PSFO, and the LFDM models indicated their high sorption affinity to the CRNPs. The adsorption of the four drugs fitted the multilayer FIM that supported the high-affinity claim. The removals of the four drugs were exothermic and spontaneous physisorption. The fabricated CRNPs possessed an excellent remediation efficiency for contaminated SW and GW; therefore, CRNPs are suggested for water remediation as low-cost sorbent.

## 1. Introduction

Worldwide, pharmaceutical contaminants (PhCs) pervade groundwater, seawater, and rivers. Perhaps it might help to clarify the critical situation of humanity that 50% of the water resources on Earth have been declared polluted since 1970 [1]. In 1999 the United States of America considered water pollution by PhCs a health threat, and more than half of China’s population was declared to be consuming contaminated drinking water in the same year [2,3,4]. The PhCs have become widespread over all of India’s rivers, groundwater, and water-treatment facilities, with up to 31.0 mg L^−1^ concentrations [5,6]. The PhCs pollution keeps increasing because of the modern life necessities for infectious treatments and agricultural, poultry, and livestock farming productivity [7,8,9]. The high-performance liquid chromatography was employed for determining chlortetracycline (CTCN) [10,11,12], tigecycline (TGCN) [13], oxytetracycline (OTCN) [14,15], and doxycycline (DXCN) [16]. In addition, these drugs have been determined via uv-vis spectrophotometry [17,18,19], and by high-performance liquid chromatography and spectrophotometry. Water contamination has a variety of sources, including runoff from agricultural fields, municipal discards, and machine-washing in pharmaceutical industries [20,21,22,23,24,25,26,27,28]. Tetracyclines (TCs) are one of the most widely used antibiotic families for treating infections, poultry and fish farms, and livestock animals. The most representative TCs includes TGCN, CTCN, OTCN, and DXCN [29]. As broad-spectrum antibiotics, TCs consumption increases as antibiotics and food preservatives increases [30,31]. Several studies exposed how TCs spread into soil and water resources in the USA, UK, and China [32,33,34,35,36,37,38]. The PhCs occurrence in water created the problem of bacterial-antibiotic resistance that is transported by genes to other microorganisms in the aquatic environment [37,39]. The therapeutics keep developing new drugs that are eventually released into the environment and intensify the same problem. This cycle of growing contamination can be stopped only via effective water treatment. The methods to remediate water with such contaminants encompass flocculation, chemical oxidation, ion exchange, and membranes. The spread of PhCs into seas, oceans, rivers, and drinking water indicates the failure of these conventional treatments [40,41,42,43,44,45,46,47,48]. Hence, developing new processes is necessary to avoid the widespread of PhCs. With the rise of nanomaterials, the PhCs removal from water by adsorption and photodegradation (PhTD) was expanded [49,50,51,52,53]. PhTD is known for its completeness as a water treatment method that requires no further steps, such as dumping or burning pollutants. Even though the PhTD was criticized for its relatively higher energy consumption and the production of harmful degradation products [50]. In addition to treating undegradable contaminants in water, such as heavy metals, the adsorption process is simpler, consumes less energy than the PhTD, and does not liberate harmful fragments [37,51,54]. Carbonaceous materials (CRMs) are traditional sorbents for removing colored compounds from solutions [55]. They are environmentally safe, easy to use, have high-surface area, and excellent adsorbing capabilities, making them a perfect candidate for water treatment [56,57,58,59,60,61,62,63].

Due to the surface area-size inverse proportionality for CRMs, this work aims to fabricate carbon nanoparticles (CRNPs). Considering the cost factor’s influence on sorbent’s applicability, the CRNPs will be prepared from waste instead of virgin precursors. Therefore, toilet tissue paper waste (TTPW) is introduced as a cheap CRNP precursor via the calcination-ball milling process. The prepared nanomaterial will be suggested as a low-cost sorbent for removing TGCN, DXCN, CTCN, and OTCN from water.

## 2. Experimental

### 2.1. Materials

Commercial toilet tissue papers were collected from the local market. Phosphoric acid 85% (H_3_PO_4_) was from Merk, Darmstadt, Germany. The TGCN was provided by the Triveni Interchem in Mumbai, India. The DXCN, CTCN, and OTCN were brought from Fluka, Bushes, Switzerland. The TTPW was collected from the trash container beside a bathroom-hand-wash site as a common source for such waste.

### 2.2. Preparation of CRNPs

For this study, 20 g of toilet paper waste TPW were carbonized at 750 °C for 2.0 h under a nitrogen gas stream (50 mL min^−1^) as inert-environment. The CRMs were placed in an autoclave, macerated in concentrated phosphoric acid, and heated at 150 °C for 2.0 h. The CRMs were washed with distilled water via vacuum filtration to remove the excess acid, then dried at 110 °C for 4.0 h. Then, 5.0 g of the CRMs were transferred to a 50 mL stainless-steel crucible with 7.0 stainless-steel balls (1 cm diameter), and the ball mill machine was set at 500 rounds per minute for 10.0 h.

### 2.3. Characterization of CRNPs

The prepared CRNPs were characterized by scanning electron energy dispersive X-ray spectroscopy (FE-SEM-EDX, JSM-IT500HR, JEOL, Tokyo, Japan) and transmission electron microscopy (JEM-1400, JEOL, Tokyo, Japan). The surface characteristics were examined by the Micromeritics surface analyzer (ASAP 2020, Norcross, GA, USA). Additionally, the prepared CRNPs were characterized via an X-ray diffractometer (Bruker, Miami, FL, USA), and the Scherrer equation (Equation (1)) was utilized for calculating the average crystal size (*D*). The lattice parameters (*a* and *c*) were computed via Equations (2) and (3), while Equation (4) was used to calculate the lattice imperfection (*ε*) [64,65].

(1) $$D=\frac{0.9\lambda }{\beta cos\theta }\;$$

(2) $$a=\frac{\lambda }{\sqrt{3}\;sin\theta }\;$$

(3) $$c=\frac{\lambda }{sin\theta }\;$$

(4) $$\varepsilon =\frac{\beta }{4\;cos\theta }$$

where *θ* is Bragg’s angle; *λ* is the source-wavelength-line (Cu-K_α_ = 1.5406 Å); *β* is the peak width at half-maximum [66].

### 2.4. Adsorption Studies

The CRNPs have been tested for removing TGCN, DXCN, CTCN, and OTCN from the aquatic environment via the batch-experiment technique. The influence of contact time and the kinetic studies were performed by stirring 120 mL of drug solution (100 mg L^−1^) with 50 mg of the CRNPs. The picked portions were filtered via a 0.22 μm syringe filter; then, the absorbance was measured in triplicate using a Shimadzu-2600i UV-Vis spectrophotometer. The adsorption capacity (*q_t_*, mg g^−1^) was computed via Equation (5). The solution pH effect on the adsorption of TGCN, DXCN, CTCN, and OTCN on CRNPs was investigated. Additionally, 100 mg L^−1^ of each drug was adjusted to different pH from 2.0 to 10.0. Due to the crucial role of the solution’s pH in the sorption process, recent studies investigated the zero charge point (pH_pzc_) for sorbents. The sorbent surface is positively charged at a pH lower than the pH_ZPC_ value and negatively charged above the pH_ZPC_. The solid addition method was employed for investigating the CRNP’s pH_pzc_, and the point in which ΔpH = 0 is the pH_pzc_ [67].

Furthermore, the contact time findings were employed to inspect the drug’s adsorption kinetics. The pseudo-first-order and pseudo-second-order models (PFOM and PSOM) illustrated in Equations (6) and (7) were used to investigate the rate of sorption. In addition, the step controlling the adsorption was explored by the intra-particle and liquid film diffusion models (IDM and LDM, Equations (8) and (9)).

(5) $$q_{t}=\frac{\left(C_{o}-C_{t}\right)\;V}{M}\;\;$$

(6) $$\ln\left(q_{e}-q_{t}\right)=\ln\left(q_{e}\right)-kt\;$$

(7) $$\frac{1}{q_{t}}=\frac{1}{k_{2}q_{e}^{2}\;t}+\frac{1}{q_{e}}\;\;$$

(8) $$q_{t}=K_{IDM}\times t^{\frac{1}{2}}+C_{i}\;$$

(9) $$\ln\left(1-F\right)=-K_{LDM}\times t\;$$

where: *C_o_*, *C_t_* (mg L^−1^) resents the drug concentrations at time zero, and *t*; *V* (L) and *M* (g) present the volume of drug solution and mass of CRNPs, respectively; *k*_1_(min^−1^): the PFOM rate constant; *k*_2_ (g mg^−1^ min^−1^): rate-constant of the PSOM; *K_IDM_* (mg g^−1^ min^−0.5^) and *K_LDM_* (min^−1^): the IDM and LDM constants; the *q_e_* (mg g^−1^) is the equilibrium adsorption capacity; *C_i_* (mg g^−1^): the boundary-layer-thickness parameter.

The initial drug concentration effect on its sorption was tested utilizing 10, 25, 50, and 100 mg L^−1^ drug solutions. Additionally, the temperature influence investigation for the adsorption of TGCN, DXCN, CTCN, and OTCN on CRNPs was performed using the different concentrations at 298, 308, and 318 °K. The obtained results were employed to study the sorption’s isotherms and thermodynamics.

### 2.5. Application of CRNPs to Natural Water Treatment

A groundwater sample (GS) was collected from Sudair industrial city (150 km north of Riyadh, Saudi Arabia), while the seawater sample (SS) was brought from the coast of Aldamam city, Saudi Arabia. A concentration of 5.0 and 10 mg L^−1^ from each drug was prepared in GS and SS, then 50 mg of the CRNPs were stirred with 120 mL of polluted water sample for 2.0 h. In order to avoid the impact of GS and SS, the standard solutions were prepared in the same matrix, and the water samples were employed as blank. The treated GS and SS samples were filtered via a membrane syringe filter (0.22 µm), and the absorbances of unabsorbed pollutants were determined by a UV-vis spectrophotometer.

## 3. Results and Discussion

### 3.1. Characterization of CRNPs

**Figure 1:**
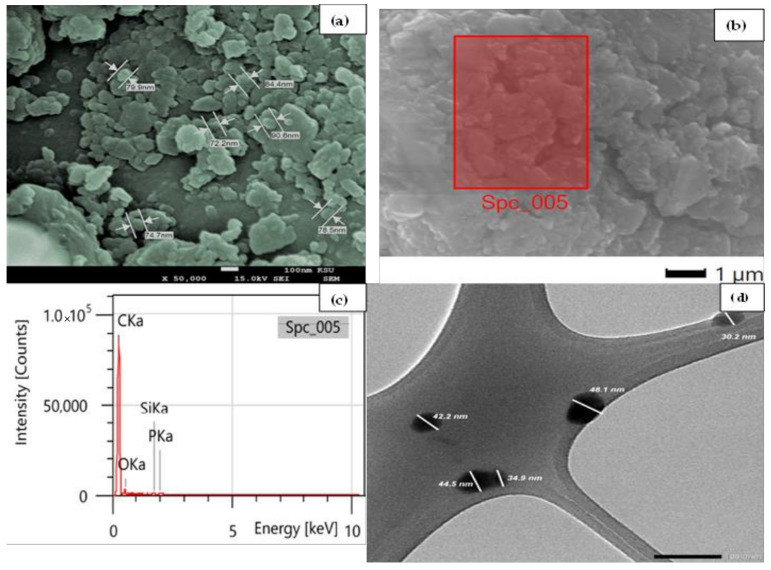
(**a**) SEM images, (**b**) electronic image from the EDX analysis, (**c**) EDX elemental results, and (**d**) TEM results for the prepared CRNPs.

The SEM results in Figure 1a revealed the CRNPs were clustered due to the ball-milling process. In addition, the CRNPs appeared with a particle size range of 72.2 to 90.6 nm, indicating a successful fabrication of nanoscale carbon particles via the suggested method. Figure 1b,c show the fabricated nanomaterial’s electronic image and the elemental composition results, respectively. The CRNPs were mainly composed of 90.6% carbon and 9.0% oxygen, while the rest of 0.4% comprised potassium and calcium traces. Additionally, the TEM analysis was employed to examine the detailed morphology, and the disunity of the CRNPs clusters revealed a particle size range of 30.2 to 48.1 nm (Figure 1d). These results were in line with the SEM results since the second may show clusters as one particle.

**Figure 2:**
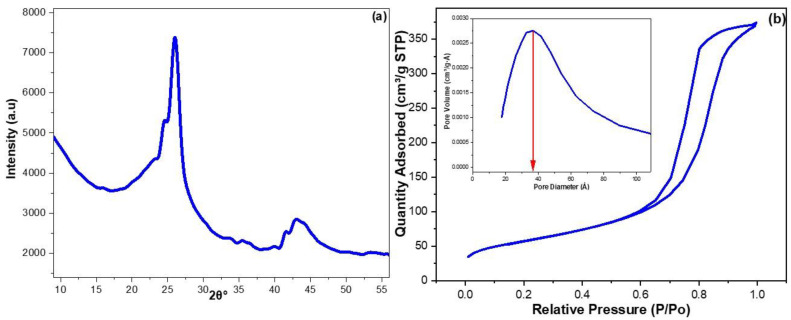
(**a**) XRD pattern for the fabricated CRNPs; (**b**) surface analysis of the CRNPs using the nitrogen adsorption-desorption isotherm.

The XRD was utilized to analyze the crystallinity and phase purity of the synthesized CRNPs (Figure 2a). The obtained results revealed diffraction peaks at 26.02 and 43.14 2θ° that can be assigned to the planes of cubical-lattice-graphite-phase of (002) and (100) [68]. Moreover, the baseline elevation around 2θ° of 10.0 indicates the occurrence of some amorphous carbon in the CRNPs. The most intense peak at 2θ° of 26.02 was employed for computing *D*, *a*, *c*, and *ε* values which were found to be 22.13 nm, 0.20 nm, 6.84 nm, and 0.38, respectively.

The surface characteristics of the synthesized CRNPs were determined via the N_2_ adsorption-desorption method (Figure 2b). The CRNPs possessed a type 2b hysteresis loop that corresponds to mesoporous materials of slit-like pores with narrow pore-neck ranges [69,70,71]. The surface area (SA) of the CRNPs was determined via Brunauer–Emmett–Teller’s (BET) procedure, whereas the pore diameter and volume (PD and PV) were estimated by Barrett-Joyner-Halenda (BJH) way. The CRNPs showed PD, PV, and SA values of 79.14 Ǻ, 0.58 cm^3^ g^−1^, and 283.04 m^2^ g^−1^, respectively; the obtained PD and PV seem suitable for the entrapment of pollutants. Additionally, these parameters were comparable to recent literature findings about CRMs [50,72,73,74].

### 3.2. Adsorption of Pharmaceutical Pollutants by CRNPs

**Figure 3:**
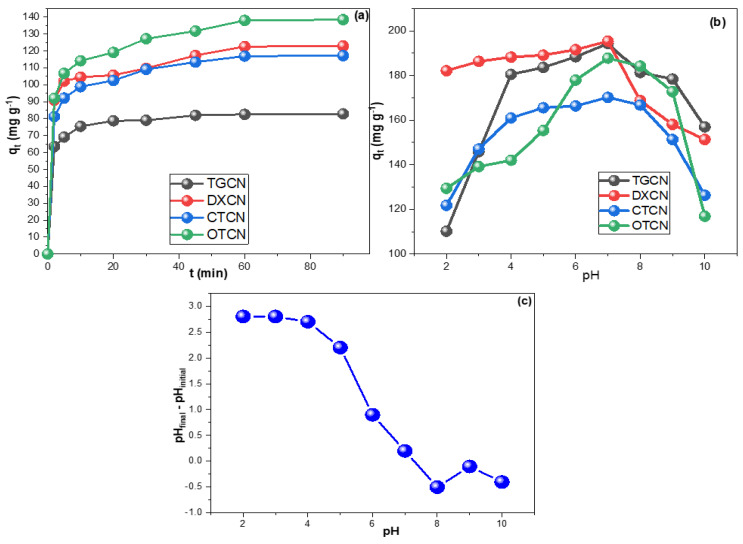
(**a**) The contact time study for the adsorption of TGCN, CTCN, OTCN, and DXCN on the synthesized CRNPs, (**b**) the influence of the solution’s pH on their sorption, and (**c**) the zero charge investigation for the fabricated CRNPs.

Figure 3a illustrates the impact of contact time on the sorption of TGCN, DXCN, CTCN, and OTCN on the CRNPs. The four drugs reached their adsorption equilibrium points within 60 min with experimental q_t_ values of 82.9, 122.8, 117.2, and 138 mg g^−1^ for TGCN, DXCN, CTCN, and OTCN, respectively. It is worth mentioning that almost 90% of these uptakes were acquired during the first 20 min of contact. Figure 3b illustrates the evaluation of pH impact on TGCN, DXCN, CTCN, and OTCN adsorptions, while Figure 3c shows the pH_ZCH_ investigation for the fabricated CRNPs. The pH_pzc_ was equal to 7.2 explains the attainment of higher *q_t_* values within the range of 6.0 to 8.0.

**Figure 4:**
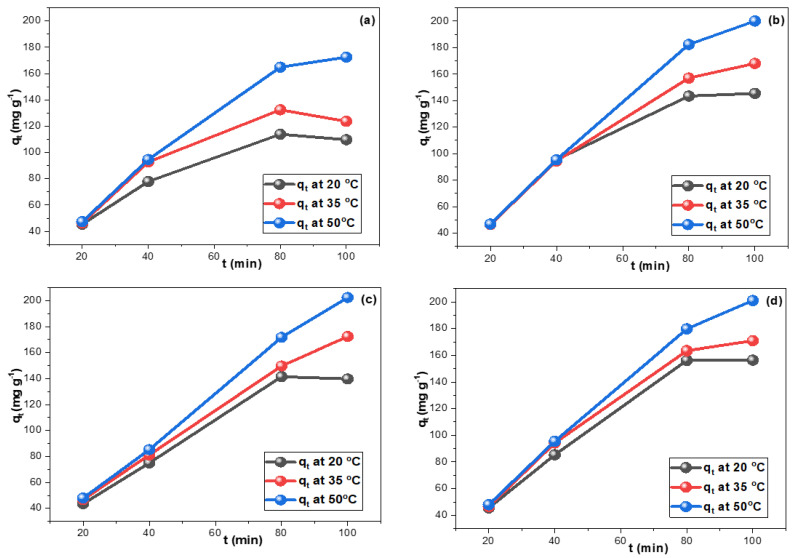
The impact of solution feeding concentration on the drug adsorption by the synthesized CRNPs at 20 °C, 35 °C, 50 °C for (**a**) TGCN, (**b**) DXCN C, (**c**) CTCN, and (**d**) OTCN.

In addition, the impact of the concentrations of the drugs on their adsorption by CRNPs was tested. Figure 4 illustrates the increase of q_t_ values as the drug concentration raised and reveals the capability of CRNPs to possess higher q_t_ values. Typically, the adsorption from 100 mg L^−1^ TGCN, DXCN, CTCN, and OTCN solutions showed q_t_ values of 172.5, 200.1, 202.4, and 200.0 mg g^−1,^ respectively. These findings inferred the applicability of the prepared CRNPs for treating industrial effluents with high pollutant concentrations. In addition, it can be concluded that the 4:5 sorbent: solution ratio is suitable for removing up to 100 mg L^−1^ pollutant concentration. The CRNPs also adsorbed the pollutants entirely from the 10 mg L^−1^ solutions, demonstrating their effectiveness in treating contaminated water resources.

### 3.3. Possible Adsorption Mechanism

**Figure 5:**
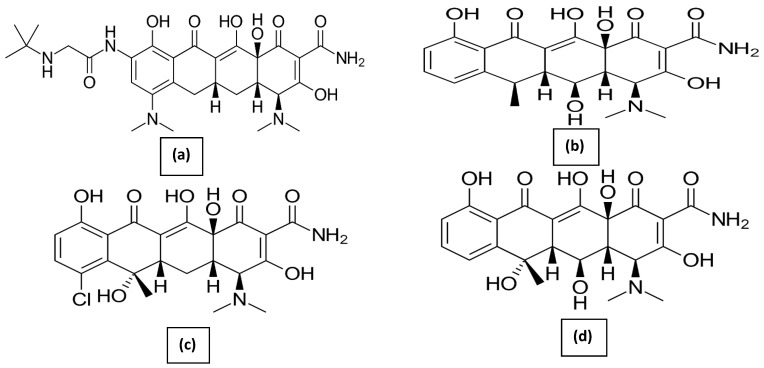
Monitored the chemical structure of (**a**) TGCN, (**b**) DXCN, (**c**) CTCN, and (**d**) OTCN.

Figure 5 monitored the chemical structure of TGCN, DXCN, CTCN, and OTCN. The heteroatoms on the drugs may contribute significantly to the adsorption through their lone pairs of electrons. The oxygen group on drugs withdraws electrons, leading to electron-deficient carbons acting as π-electron-acceptors; meanwhile, the high electron density on CRNPs oxygenated groups will be the π-electron-donor. The multi-carbonyl groups of these drugs would be protonated in acidic media (pH < 6.0), and the cationic-formed sites may repel each other and prevent multilayer sorption. In contrast, in an alkaline media of (pH > 8), the hydroxyl groups may compete with the drugs on the sorbent sites, and the deprotonate hydroxyl groups of a drug may increase the repulsion [75,76].

### 3.4. Adsorption Kinetics

**Figure 6:**
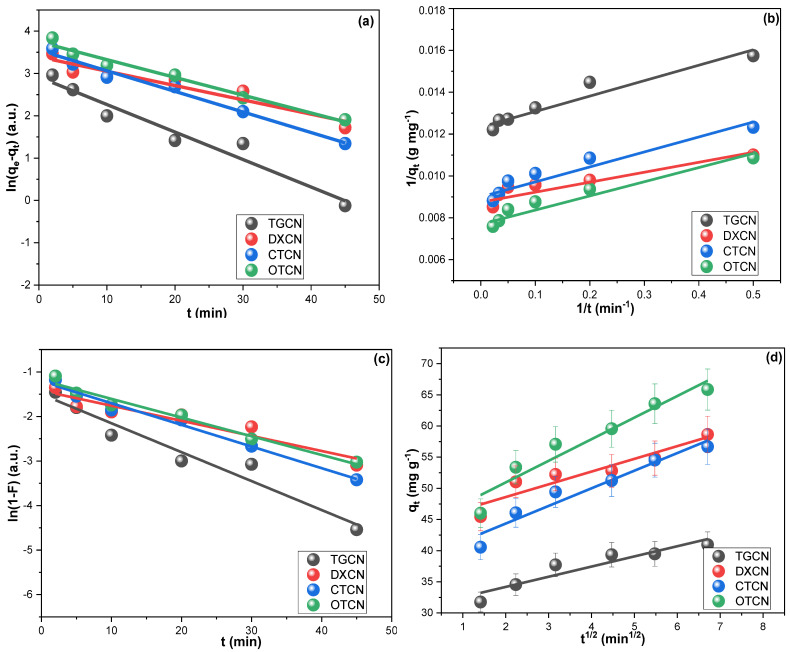
(**a**) PSFOM, (**b**) PSSOM (**c**) LFD, (**d**) IPD investigations for the adsorption of TGCN, DXCN, CTCN, and OTCN on the synthesized CRNPs from 100 mg L^−1^ solutions at 25 °C.

The linear regression plots of the PSFOM, PSSOM, IDM, and LDM for TGCN, DXCN, CTCN, and OTCN sorption on the CRNPs are illustrated in Figure 6. The *k*_1_, *k*_2_,* K_IDM,_* and *K_LDM_* values gathered in Table 1 were computed utilizing the extracted regression parameters (slope and intercept) [77]. The results revealed that TGCN, DXCN, CTCN, and OTCN sorption fitted the PSFOM model, which may justify their relatively rapid equilibrium. Furthermore, the investigation of the rate-control step revealed that the intraparticle diffusion step controlled the adsorption of DXCN, CTCN, and OTCN on the CRNPs. Conversely, the film-diffusion step was the slowest step during TGCN adsorption. These findings indicated that TGCN has a higher affinity toward the CRNPs surface than the other three drugs and implied the significance of pore-diffusion in removing DXCN, CTCN, and OTCN [78].

### 3.5. Adsorption Isotherms

**Figure 7:**
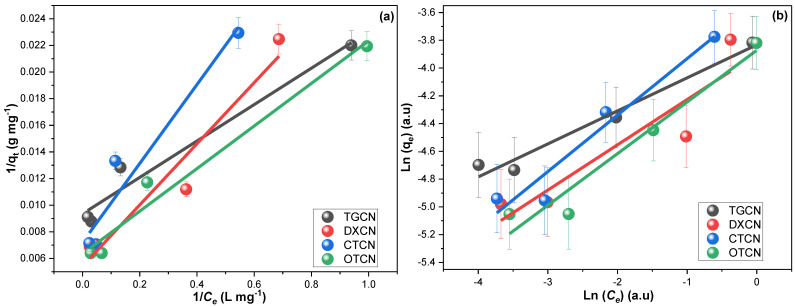
The (**a**) LIM and (**b**) FIM investigations for the adsorption of TGCN, DXCN, CTCN, and OTCN on the synthesized CRNPs using 10, 25, 50, and 100 mg L^−1^ drug solutions at 20, 35, and 50 °C.

The Langmuir (LIM, Equation (7)) and Freundlich models (FIM, Equation (8)) were the most used isotherm models for describing adsorption processes. Both models were utilized to inspect TGCN, DXCN, CTCN, and OTCN sorption on the fabricated CRNPs via their linearized forms (Equations (7) and (8)).

(10) $$\frac{1}{q_{e}}=\frac{1}{K_{l}\;q_{m}}\times \frac{1}{C_{e}}+\frac{1}{K_{l}}$$

(11) $$\ln q_{e}=\ln K_{f}+\frac{1}{n}\;\ln C_{e}$$

where *K_l_* (L mg^−1^) is LIM constant, *K_f_* (L mg^−1^) is FIM constant; *C_e_* (mg L^−1^) is the equilibrium solution concentration, *q_m_* is the computed-maximum-sorption capacity, while the 1/*n* is Freundlich-adsorption-intensity [79]. Figure 7 illustrates the linear fittings of LIM and FIM investigations for TGCN, DXCN, CTCN, and OTCN sorption on the fabricated CRNPs.

The findings monitored in Table 2 revealed better fitting to LIM. The 1/*n* values of less than unity for the four drugs indicated that their sorption were favorable [80]. Furthermore, the fitting of TGCN, DXCN, CTCN, and OTCN sorption to the LIM was in line with their PFOM agreement [81,82].

### 3.6. Adsorption Thermodynamics

**Figure 8:**
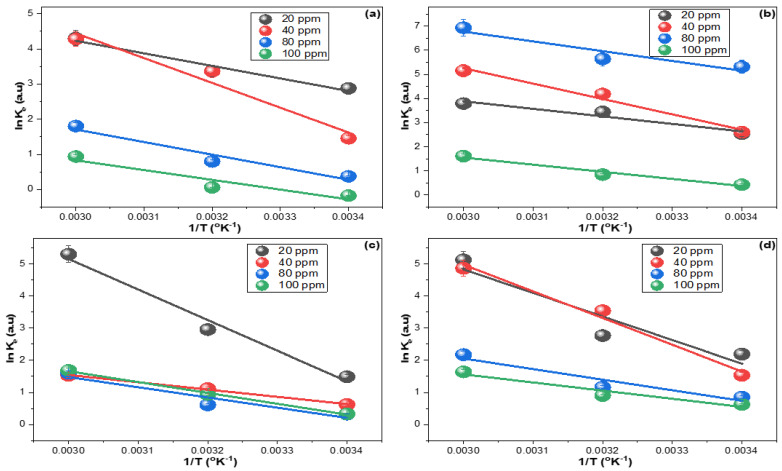
Thermodynamics study of the sorption of (**a**) TGCN, (**b**) DXCN, (**c**) CTCN, and (**d**) OTCN on the synthesized CRNPs at 298, 313, and 328 °K by utilizing 20, 40, 80, and 100 mg L^−1^ as fed concentrations.

The thermodynamics of TGCN, DXCN, CTCN, and OTCN removal by the CRNPs were inspected. The slope and intercept extracted from the plot of Equation (12) (Figure 8) were utilized in computing the enthalpy and entropy (Δ*S*° and Δ*H*°). The Gibbs free energy (Δ*G*°) was obtained by applying the Δ*S*° and Δ*H*° values in Equation (13). The ideal gas constant (R) was used as 0.0081345 kJ mol^−1^ for calculating these parameters, and the findings are in Table 2.

(12) $$\ln K_{c}=\frac{\Delta H{}^{\circ}}{RT}+\frac{\Delta S{}^{\circ}}{R}\;$$

(13) Δ G°=Δ H° − T Δ S°

The spontaneity and exothermic nature can be noticed for TGCN, DXCN, CTCN, and OTCN adsorptions from their negative Δ*G*° values (Table 2), and their negative Δ*H*° values supported the exothermic nature claim. Additionally, the average Δ*H*° values for the different concentrations were below 80.0 kJ mol^−1^, indicating that CRNPs removed TGCN, DXCN, CTCN, and OTCN via physisorption [83,84,85,86,87].

### 3.7. Natural Water Treatment and Regeneration Investigations

**Figure 9:**
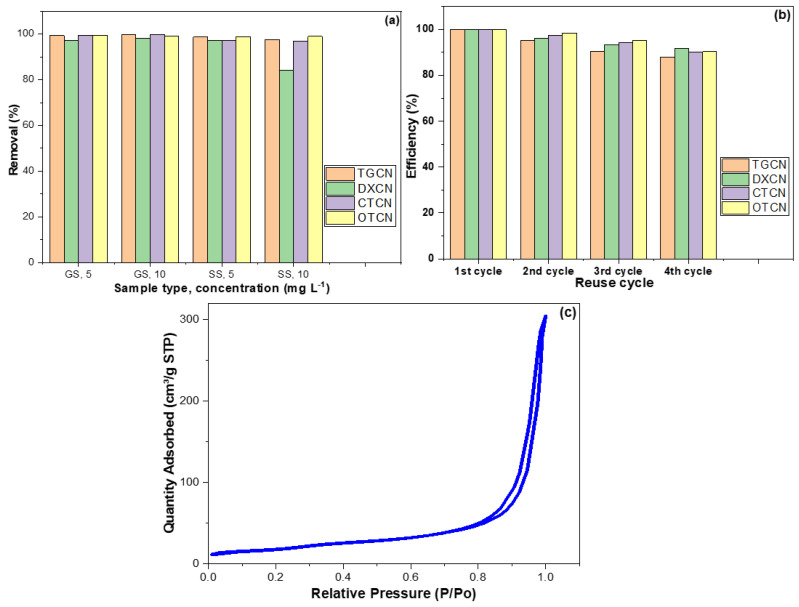
(**a**) Adsorption of TGCN, DXCN, CTCN, and OTCN on CRNPs from contaminated GS and SS; (**b**) the removal efficiency for TGCN, DXCN, CTCN, and OTCN by the regenerated CRNPs. (**c**) The surface area reduction to 200 m^2^ g^−1^ for the regenerated CRNPs.

The CRNPs were tested for removing TGCN, DXCN, CTCN, and OTCN from synthetically polluted GS and SS. The average removal percentage for TGCN, DXCN, CTCN, and OTCN were 98.9%, 94.3%, 98.4%, and 99.1%, respectively, with RSD values of 0.894%, 7.192%, 1.413%, and 0.245%, respectively. Although the fabricated CRNPs were excellent in removing these pollutants from both water types (Figure 9a), it can be seen that the remediation of GS was better than the SS, which indicates that the CRNPs can be employed for the sorption of metal ions. This result can be attributed to salt concentration (mainly saline) in the SS, which may affect the pollutant’s migration to the CRNPs surface.

Additionally, the reusability of the CRNPs in removing TGCN, DXCN, CTCN, and OTCN was investigated. According to the pH study, the CRNPs used with DXCN and OTCN were regenerated by sonication for 20 min with 20 mL of 1.0 M sodium hydroxide (NaOH). The sonication was repeated with 10 mL ethanol, then filtered, rinsed with distilled water, and dried at 105 °C for 2.0 h. On the other hand, CRNPs used with TGCN and CTCN were regenerated in the same manner but substituting the 1M NaOH with 1M hydrochloric acid. The performance of virgin CRNPs was considered 100% efficient, and the subsequent cycles were relatively determined (Figure 9b) [51,88]. The regenerated CRNPs showed mean efficiency of 93.40%, 95.31%, 95.49%, and 95.94% for TGCN, DXCN, CTCN, and OTCN, respectively, with RSD% values of 5.71, 3.78, 4.42, and 4.46. According to the kinetic studies, the LFDM was the slowest sorption step indicating the ease of drug penetration into the CRNP’s inner shells. The surface area reduction to 200 m^2^ g^−1^ for the regenerated CRNPs (Figure 9c) can be attributed to the entrapment of some molecules in the sorbent’s pores, which may explain the efficiency decrease inversely with the regeneration round number.

## 4. Conclusions

CRMs have been derived from TPW via thermal carbonization. A concentrated phosphoric acid was employed for pores opening, and the ball-milling process was utilized to down-size the CRMs and fabricate CRNPs. The batch adsorption experiments were followed to investigate the usability of CRNPs for removing TGCN, DXCN, CTCN, and OTCN from water. The agreement of TGCN, DXCN, CTCN, and OTCN sorption to the PSFO and the LFDM models indicated high adsorption affinity to the CRNPs. Furthermore, the equilibrium results for the four drugs being fitted to the multilayer FIM supported the high-affinity claim. The removal appeared to be exothermic and spontaneous physisorption for the four drugs. Although its efficiency decreased slightly during the consecutive reuses, the CRNPs possessed an excellent remediation efficiency for SS and GS contaminated by TGCN, DXCN, CTCN, and OTCN; Therefore, the prepared CRNPs are recommended for groundwater and seawater remediation as a low-cost sorbent.

## Figures and Tables

**Table 1:** The kinetic results of the adsorption of TGCN, DXCN, CTCN, and OTCN on the fabricated CRNPs from 50 mg L^−1^ solutions at 25 °C.

Adsorption Kinetic								
Adsorption rate order								
PSFOM					PSSOM			
Parameter	TGCN	DXCN	CTCN	OTCN	TGCN	DXCN	CTCN	OTCN
*q_e_* exp. (mg g^−1^)	82.80	122.87	117.21	138.44	82.80	122.87	117.21	138.44
*q_e_* cal. (mg g^−1^)	18.437	29.714	34.765	42.360	81.037	114.286	111.111	130.039
*R* ^2^	0.951	0.910	0.981	0.974	0.930	0.809	0.904	0.912
Rate constant	0.065	0.034	0.049	0.042	0.012	0.009	0.009	0.008
Adsorption mechanism								
Pollutant	IDM					LDM		
	*K_IP_* (mg g^−1^ min^0.5^)		*C* (mg g^−1^)		*R* ^2^	*K_LF_* (min^−1^)	*R* ^2^	
TGCN	1.632		30.916		0.884	0.068	0.976	
DXCN	2.043		44.514		0.887	0.064	0.818	
CTCN	2.828		38.692		0.942	0.071	0.913	
OTCN	3.466		44.019		0.932	0.068	0.877	

**Table 2:** The isotherms and thermodynamic results for the adsorption of TGCN, DXCN, CTCN, and OTCN on the synthesized CRNPs.

Adsorption Isotherms						
Isotherm model	LIM			FIM		
Drug ↓	*R*^2^ (a.u.)	*K_L_* (L mg^−1^)	*q_m_* (mg g^−1^)	*R*^2^ (a.u.)	*K_f_* (L mg^−1^)	*n*^−1^ (a.u.)
TGCN	0.974	106.925	0.684	0.972	0.022	0.238
DXCN	0.944	184.213	0.236	0.848	0.326	0.311
CTCN	0.937	138.692	0.243	0.943	0.029	0.407
OTCN	0.973	157.557	0.397	0.949	0.021	0.372
Thermodynamic parameters						
Initial conc. (mg L^−1^)	Δ*H*° (k Jmol^−1^)	Δ*S*° (kJ mol^−1^)	Δ*G*° (kJ mol^−1^) 298 K	Δ*G*° (kJ mol^−1^) 308 K	Δ*G*° (kJ mol^−1^) 318 K	
TGCN						
20	38.451	0.152	−6.931	−9.215	−11.499	
40	76.793	0.271	−3.949	−8.014	−12.078	
80	38.448	0.131	−0.676	−2.646	−4.615	
100	29.776	0.098	0.700	−0.764	−2.227	
DXCN						
20	34.002	0.136	−6.498	−8.536	−10.575	
40	69.313	0.255	−6.642	−10.465	−14.288	
80	43.464	0.189	−12.751	−15.581	−18.410	
100	31.822	0.110	−0.910	−2.558	−4.205	
CTCN						
20	102.717	0.356	−3.276	−8.611	−13.947	
40	24.608	0.088	−1.556	−2.873	−4.190	
80	34.168	0.116	−0.501	−2.246	−3.991	
100	36.517	0.125	−0.743	−2.619	−4.494	
OTCN						
20	78.631	0.280	−4.673	−8.866	−13.059	
40	90.231	0.316	−4.015	−8.758	−13.502	
80	35.365	0.125	−1.819	−3.691	−5.562	
100	27.247	0.096	−1.353	−2.792	−4.232	
